# Feature extraction based on microstate sequences for EEG–based emotion recognition

**DOI:** 10.3389/fpsyg.2022.1065196

**Published:** 2022-12-23

**Authors:** Jing Chen, Zexian Zhao, Qinfen Shu, Guolong Cai

**Affiliations:** ^1^School of Computer Science and Engineering, Xi’an University of Technology, Xi’an, China; ^2^Faculty of Computing, Harbin Institute of Technology, Harbin, China; ^3^Department of Neurology, Zhejiang Hospital, Hangzhou, China; ^4^Department of Critical Care Medicine, Zhejiang Hospital, Hangzhou, China

**Keywords:** feature extraction, EEG, emotion recognition, microstate analysis, k-mer frequency

## Abstract

Recognizing emotion from Electroencephalography (EEG) is a promising and valuable research issue in the field of affective brain-computer interfaces (aBCI). To improve the accuracy of emotion recognition, an emotional feature extraction method is proposed based on the temporal information in the EEG signal. This study adopts microstate analysis as a spatio-temporal analysis for EEG signals. Microstates are defined as a series of momentary quasi-stable scalp electric potential topographies. Brain electrical activity could be modeled as being composed of a time sequence of microstates. Microstate sequences provide an ideal macroscopic window for observing the temporal dynamics of spontaneous brain activity. To further analyze the fine structure of the microstate sequence, we propose a feature extraction method based on k-mer. K-mer is a k-length substring of a given sequence. It has been widely used in computational genomics and sequence analysis. We extract features that are based on the $D_{2}^{*}$ statistic of k-mer. In addition, we also extract four parameters (duration, occurrence, time coverage, GEV) of each microstate class as features at the coarse level. We conducted experiments on the DEAP dataset to evaluate the performance of the proposed features. The experimental results demonstrate that the fusion of features in fine and coarse levels can effectively improve classification accuracy.

## Introduction

Emotions are responses to significant internal and external events (Salas et al., 2012). Emotion plays a crucial role in rational decision-making, perception, and human intelligence. Meanwhile, emotional intelligence is essential in human-machine interactions. It is the ability to identify, assess, understand, and manage the emotions of humans. Emotion states can be detected from the face, voice, and physiological signals. With the rising interest in brain-computer interaction, recognizing emotion from Electroencephalography (EEG) is a promising and valuable research issue. EEG is a method to record an electrogram of the electrical activity on the scalp. EEG is non-invasive and can be applied to healthy people and patients with no risk. It has been widely used to investigate the neural correlates of emotions (Lin et al., 2010; Daly et al., 2014; Balasubramanian et al., 2018; Hsu et al., 2018). Emotion recognition based on EEG has received increased attention in the field of affective computing.

Many features of EEG-based emotion recognition have been studied in the past (Song et al., 2018; Suhaimi et al., 2020; Tao et al., 2020; Torres et al., 2020; Chen et al., 2022). To compare the existing features systematically, Jenke et al. (2014) reviewed feature extraction methods for emotion recognition from EEG based on over 30 studies. These features are commonly distinguished in the time domain, frequency domain, and time-frequency domain. More recently, considering the high temporal resolution of EEG, microstate analysis has been adopted to understand the spatial–temporal dynamics of complex brain activities (Michel and Koenig, 2018). Microstate analysis treats multichannel EEG as a limited number of microstate classes. These EEG microstates can reflect the dynamic process of a variety of cognitive states and traits. Gianotti et al. (2008) studied the temporal dynamics of the neural activity that responded to emotional words and picture stimuli using ERP microstate analysis. Shen et al. (2020) explored EEG microstates for emotional experiences during music video watching. Hu et al. (2022) systematically examined and compared the microstates for task-state EEG analysis during naturalistic music videos. In the existing studies, most studies acknowledge four standard microstate maps that can explain up to 65–85% of the EEG signal’s global variance. Several studies also suggested that the number of microstate classes was explicitly driven by the data (Muthukrishnan et al., 2016; D’Croz-Baron et al., 2019).

After microstate classes are identified, the original individual EEG data can be labeled as a microstate sequence by fitting back these microstate classes to topographies at the sample point. The EEG microstate sequences (EEG-MS) are symbolic time series related to potential neurophysiological relevance. Previous researchers have proposed several temporal parameters to analyze the EEG-MS, e.g., duration, occurrence, time coverage, and transition probabilities. These parameters of microstate sequences have been proven to offer potential biomarkers for some diseases, such as mood and anxiety disorders(Al Zoubi et al., 2019), autism spectrum disorder (D’Croz-Baron et al., 2019), and schizophrenia(Kim et al., 2021). These parameters represent the overall characteristics of MS. However, there is a need for further refinement and exploration of MS at a finer level.

In the field of computational genomics and sequence analysis, comparative analysis for RNA/DNA sequencing data has been studied for decades. K-mer is an important concept in comparative analysis. K-mer is the substring of length k contained within a biological sequence. The frequency of a set of k-mer can be used as a signature of the underlying sequence. Kirk et al. (2018) developed the k-mer sequence comparison method to deconstruct linear sequence relationships in IncRNAs. They found that k-mer-based classification is a powerful approach to detect relationships between sequence and function. Moreover, some statistics of k-mer were studied to estimate the genetic similarity (Murray et al., 2017; Deorowicz, 2020). Wen et al. (2014) proposed a k-mer natural vector model based on the distributions of k-mer in the genetic sequence.

**Figure 1:**
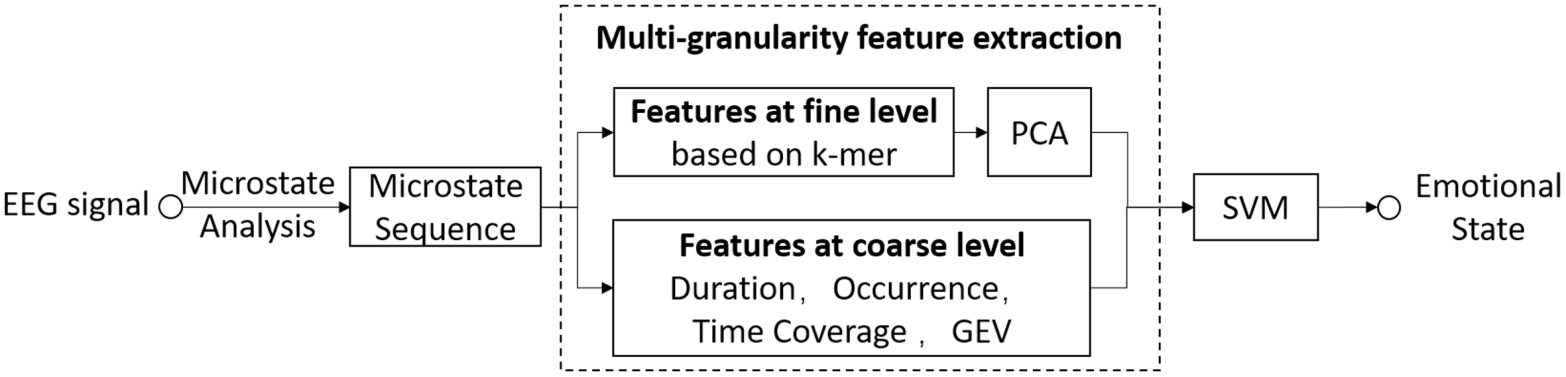
The schema of the methodology.

As EEG-MS are symbolic time series that is similar to DNA/RNA sequence, we adopt the concept of k-mer to discover the finer characteristics of the EEG-MS. In this paper, we propose a feature extraction method at fine and coarse levels for emotion recognition based on EEG-MS (as Figure 1). The features at the fine level are extracted based on the statistics of k-mer. In addition, we also extract four parameters (duration, occurrence, time coverage, GEV) of each microstate class as features at the coarse level. We fuse these features to improve the performance of emotion recognition from EEG signals.

## Materials and methods

Our method can be summarized as follows. We start by generating the microstate sequences using microstate analysis. We then use k-mer frequency analysis to further investigate the relationship between emotional states and the microstate sequences. Finally, we extracted features based on k-mer to estimate emotion based on EEG. We now describe each step in detail.

### Microstate analysis

EEG microstate was first introduced by Lehmann et al. (1987). They observed that the ongoing EEG was comprised of a discrete set of a few prototypical topographies that remained stable for around 80–120 ms before rapidly transitioning to a different topography. These periods of quasi-stable EEG topography have been called microstates. Microstate analysis mainly consists of two stages: (1) segmentation of EEG data to find the most representative template maps, which correspond to the different microstate classes, and (2) fitting these classes back to the EEG data to get the microstate sequences.

The segmentation stage is carried out by running a two-step cluster analysis. For each participant, the global field power (GFP) of each trial is calculated. GFP represents the global pattern of brain activity and is defined as follows:

(1) $$GFP\left(t\right)=\sqrt{\frac{\sum_{i=1}^{N}{\left(u_{i}\left(t\right)-\bar{u\left(t\right)}\right)}^{2}}{N}}$$

where *N* denotes the number of electrodes, $u_{i}\left(t\right)$ is the measured voltage of a specific electrode at time *t*, $\;\bar{u\left(t\right)}$ is the average voltage of the *N* electrodes at the respective sample time *t*. The topographies around peaks of the GFP are considered with the optimal signal-to-noise ratio. We smooth GFP with a Gaussian-weighted moving average of 50-time points. EEG topographies at the smoothing GFP peaks are selected to conduct further clustering. They are submitted to a cluster analysis using dual threshold-based Atomize and Agglomerate Hierarchical Clustering (DTAAHC) to identify the template microstates (Chen et al., 2021). DTAAHC is a bottom-up hierarchical clustering. The advantages of DTAAHC are the automatic identification of the optimal microstate classes and less computational cost. The optimal number of microstate classes is determined automatically according to the global explained variance (GEV) and the global map dissimilarity (GMD). The GEV measures the percentage of data explained by microstate classes. The GMD measures the topographic differences of microstate classes. The microstates are expected to be distinct and could explain more original topographies. The clustering analysis is first done at the individual level. Then the template maps for every single subject were submitted to a second DTAAHC cluster to identify the most dominant clusters across all subjects.

In the back-fitting stage, each time frame (or sample point) of the original EEG data is assigned to one specific microstate. Specifically, we calculate the spatial correlation between the instantaneous EEG topography and each microstate class. Each sampling point is labeled according to the microstate with the greatest correlation. To keep the microstate label stable, temporal smoothing is applied to avoid interruptions of noise. In the fitting process, we set temporal smoothing parameters [strength = 10, window half-size = 3 (Pascual-Marqui et al., 1995)].

### K–mer frequency analysis

#### K–mer

*K*-mer frequency analysis is originally used in computational genomics and sequence analysis. *K*-mer is a *k*-length substring of a given sequence. *K*-mer counting is to determine the occurrences of *k*-mer in a DNA/RNA sequence or sets of sequences (Marçais and Kingsford, 2011). *K*-mer frequency is an essential and crucial feature used in biological sequences. It is used to reveal the genetic characteristics of biological sequences and measure the similarity between sequences. Similar to DNA/RNA sequences, microstate sequences are symbolic time series. *K*-mer frequency can also be used as a “signature” of the unique microstate sequence.

**Figure 2:**
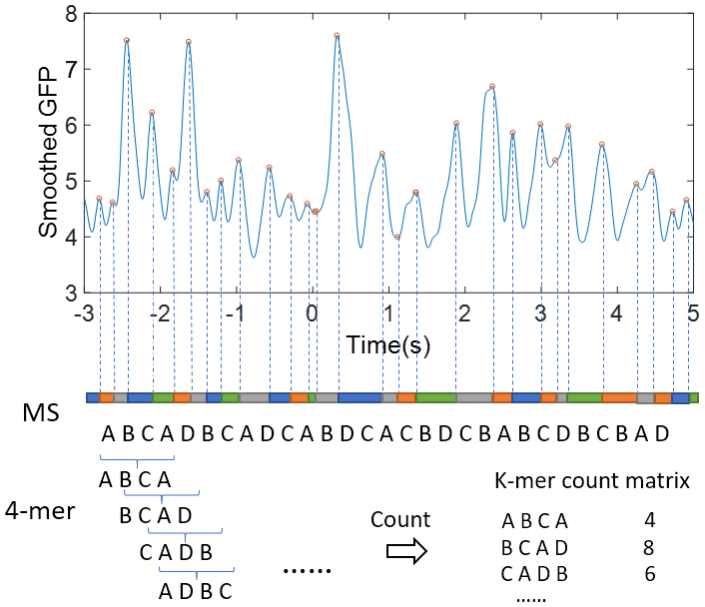
Example of *k*-mer on microstate sequence (MS).

Given a microstate sequence $X=\left\{x_{1},x_{2},\ldots ,x_{n}\right\}$ of length *n*, the sequence consists of *m* microstate categories $\Omega =\left\{y_{1},y_{2},\ldots ,y_{m}\right\}$ . For fixed-length *k* > 0, a *k*-mer is a subsequence of length *k*. Since there are *m* microstates, one can construct a total number of $m^{k}$ possible *k*-mer. Specifically, a counter of length *k* moves along the sequence and it will count the signature of a *k*-mer. Thus, it will count a total number of n−k+1 over the sequence *X*. Therefore, the occurrence frequency of each *k*-mer is denoted as:

(2) $$f_{i}=\frac{Occ_{i}}{n-k+1}\;i\in \left[1,m^{k}\right]$$

where $Occ_{i}$ is the number of occurrences of the *i*th *k*-mer. Figure 2 illustrates an example of 4-mer counting for a microstate sequence.

#### The probability of k-mer

The microstate sequences can be modeled as r-order homogeneous stationary Markov chains. The probability of the occurrence of a specific microstate at a position in a sequence depends on the proceeding r microstates. Considering a *k*-mer $w=w_{1}w_{2}\ldots w_{k}$ , the probability of *w* can be expressed with the first-order Markov chain, as follows:


(3) $$p\left(w\right)=p\left(w_{1}\right)\times \overset{k}{\underset{j=2}{\prod }}T\left(w_{j-1},w_{j}\right)$$

where $w_{j-1}$ and $w_{j}\;$ represent the (j−1) th and the *j*th characters of the *k*-mer *w*, $p\left(w_{1}\right)$ represents the probability of the occurrence of its first character, and $T\left(w_{j-1},w_{j}\right)$ represents the transition probability from $w_{j-1}$ to $w_{j}$ .

#### Sequence comparisons

*K*-mer plays an increasingly important role in rapid sequence comparison. If two sequences are closely related, the *k*-mer contents of both sequences are expected to be very similar. One of the most widely used statistics for sequence comparison based on *k*-mer is the $D_{2}$ . It is one of the most intuitive ways to find the similarity between two sequences. In this work, we explore the similarity between microstate sequences based on $D_{2}$ statistic. More specifically, suppose that two microstate sequences, $X=\left\{x_{1},x_{2},\ldots ,x_{n}\right\}$ and $Y=\left\{y_{1},y_{2},\ldots ,y_{m}\right\}$ , are independent. Let $X_{w}$ and $Y_{w}$ denote the number of occurrences of *k*-mer *w* in *X* and *Y*, respectively. The $D_{2}$ is defined by

(4) $$D_{2}=\underset{w\in A}{\sum }X_{w}Y_{w}$$

where A is the space of all possible *k*-mer. However, this statistic is found to be dominated by background noise in the non-uniform case (von Wegner et al., 2017). In the work of (Reinert et al., 2009; Wan et al., 2010), they proposed a self-standardized version of $D_{2}$ , named $D_{2}^{*}\;$ . The centralized count variable is denoted as

(5) $${\tilde{X}}_{w}=X_{w}-\left(n-k+1\right)p{\left(w\right)}_{X}$$

(6) $${\tilde{Y}}_{w}=Y_{w}-\left(m-k+1\right)p{\left(w\right)}_{Y}$$

where *n* and *m* are the length of sequence *X* and *Y*, respectively. *k* is the length of *k*-mer. $\;p{\left(w\right)}_{X}$ and $p{\left(w\right)}_{Y}$ is the probability of *w* in sequence *X* and *Y*, respectively.

The $D_{2}^{*}$ statistic is given by

(7) $$D_{2}^{*}=\underset{w}{\sum }\frac{{\tilde{X}}_{w}{\tilde{Y}}_{w}}{\sqrt{\left(n-k+1\right)p{\left(w\right)}_{X}}\sqrt{\left(m-k+1\right)p{\left(w\right)}_{Y}}}$$

The normalized $D_{2}^{*}$ dissimilarity is

(8) $$D_{2}^{*}\_norm=1-\frac{D_{2}^{*}}{\sqrt{\begin{array}{l} \sum_{w}\;{\tilde{X}}_{w}{}^{2}/\\ \left(\left(n-k+1\right)p{\left(w\right)}_{X}\right) \end{array}}\sqrt{\begin{array}{l} \sum_{w}\;{\tilde{Y}}_{w}{}^{2}/\\ \left(\left(m-k+1\right)p{\left(w\right)}_{Y}\right) \end{array}}}$$

### Emotion recognition based on EEG

#### Proposed features

Microstate sequences are symbolic time series related to potential neurophysiological relevance. In this study, we extract features from the series at fine and coarse levels.

At the coarse level, four conventional temporal parameters per microstate are extracted: duration, occurrence, time coverage, and GEV. The duration is the average time that a given microstate was uninterruptedly present. The occurrence is the mean number of a given microstate per second. The time coverage is the percentage of analysis time covered by a given microstate. The GEV is the percentage of original data that can be explained by a given microstate. These statistical parameters mainly represent the overall temporal characteristics of microstate sequences.

At the fine level, we extracted features based on *k*-mer. The feature for each *k*-mer is calculated as

(9) $$F_{w}=\frac{{\tilde{X}}_{w}}{\sqrt{\left(n-k+1\right)p{\left(w\right)}_{X}}}$$

Here we set $\frac{0}{0}=0$ . n is the length of the microstate sequence X . $p{\left(w\right)}_{X}$ is the probability of *w* in sequence *X.* This probability can be calculated under 1-order Markov chains. $\tilde{X_{w}}$ is derived from equation 5.

#### Dimension reduction

If there are *m* basis of microstates, one can construct a total number of $m^{k}$ possible *k*-mer. Therefore, the feature vector is high-dimensional and redundant. Principal component analysis (PCA) is a technique for reducing the dimensionality of such datasets. PCA is the process of computing the principal components and using them to perform a change of basis on the data, sometimes using only the first few principal components and ignoring the rest. It is defined as an orthogonal linear transformation that transforms the data to a new coordinate system such that the greatest variance by some scalar projection of the data comes to lie on the first coordinate (called the first principal component), the second greatest variance on the second coordinate, and so on.

#### Classification

SVM is a supervised machine learning model and has become a popular tool in time series forecasting. The advantages of SVM are good generalization performance, the absence of local minima, and the sparse representation of the solution. The basic idea of SVM is to construct the optimal marginal hyperplane iteratively to minimize errors. In this study, we use SVM to predict emotion states from EEG signals.

## Experimental results

### Music–evoked EEG dataset

In this research, we use the popular public database, Dataset for Emotion Analysis using Physiological signals (DEAP), to analyze affective states (Koelstra et al., 2011). DEAP is a multimodal dataset, including EEG, MEG, Galvanic skin resistance (GSR), electrooculography (EOG), blood volume pressure, skin temperature, and respiration pattern. We use 32-channel EEG original signals for emotion recognition based on microstate analysis. The raw EEG data can be downloaded from the following http://www.eecs.qmul.ac.uk/mmv/datasets/deap/.

These EEG data are collected from 32 subjects. During the experiment, 40 music videos (1 min) are used as the stimulus to elicit emotions for each subject. Before each video is displayed, a 5 s baseline is recorded. Each participant is requested to finish a self-assessment foFr arousal, valence, and dominance on a scale of 1 to 9 after watching.

### Microstates for music–evoked emotion

#### Microstate classes

Before identifying the microstates, the EEG data should be preprocessed. More specifically, the EEG data is average referenced, down-sampled to 128 Hz, filtered with a 1–45 Hz cutoff, and removed eye artifacts with ICA. The 5 s baseline before stimuli is used to correct data for stimulus-unrelated variations.

**Figure 3:**
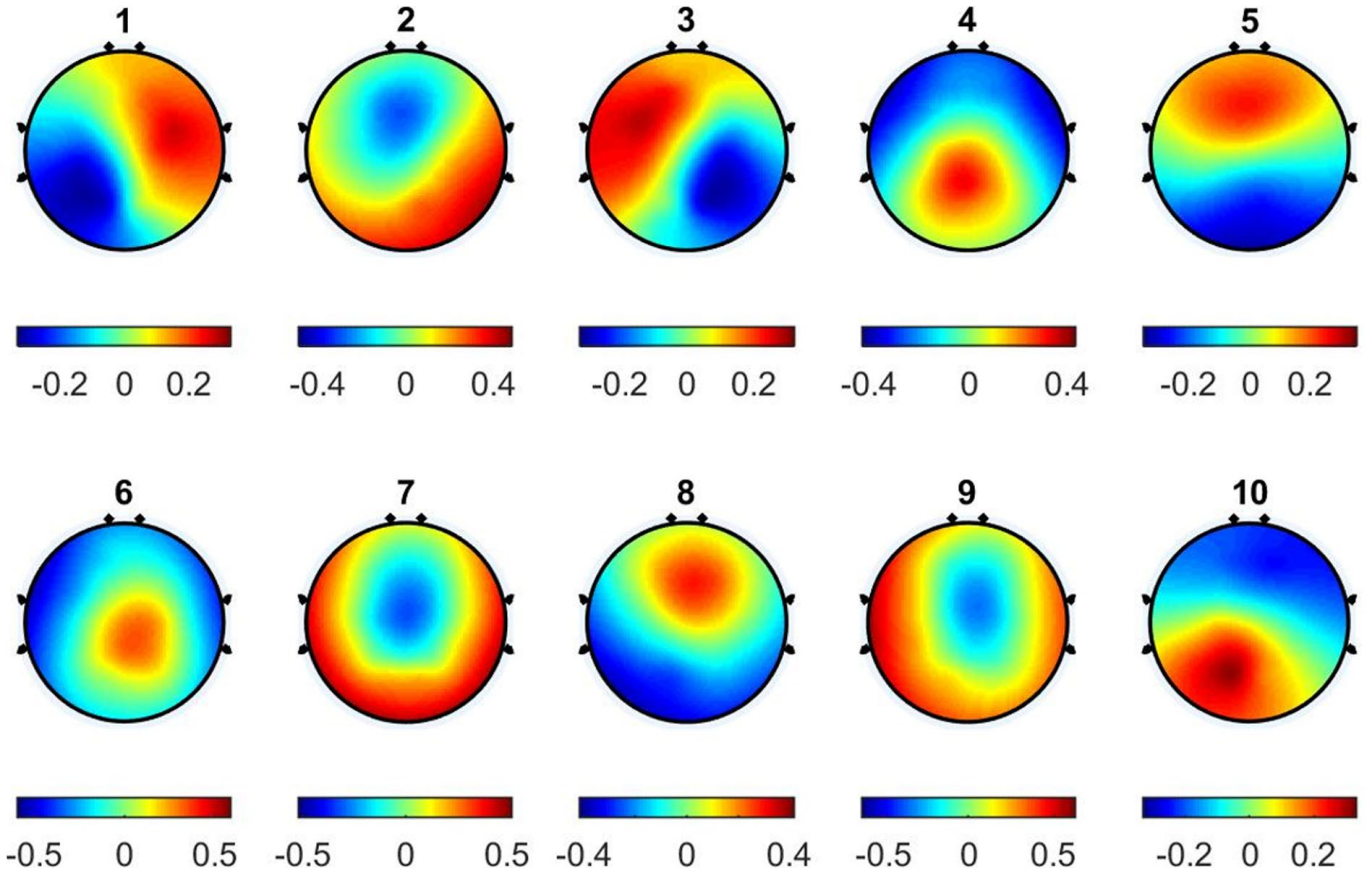
The topographical maps of the microstates across subjects.

During the cluster analysis using DTAAHC, the thresholds of global explained variance (GEV) and global map dissimilarity (GMD) are set to 85% and 0.1, respectively. We identify 10 microstates for music-evoked emotional EEG. These microstates explain 86% of the variance of all global field power peaks. And the GMD across different microstates is less than 0.1. Figure 3 illustrates 10 microstate topographies.

#### K–mer frequency

**Figure 4:**
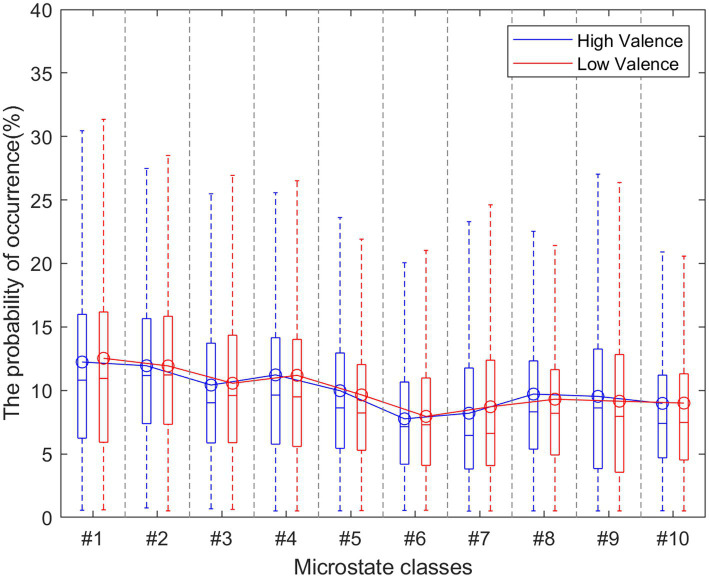
The probability of the occurrence of a specific microstate.

The original individual EEG data are labeled as a microstate sequence (MS) with fitting back 10 microstate classes to topographies at the sample point. Considering the valence dimension, the dataset is separated into high vs. low valence groups. The ratings of valence higher than 4.5 are high valence levels and vice versa. Among the total 1,280 trials of all subjects, 736 trials are labeled as high valence and 544 as low valence. Figure 4 presents the probability of the occurrence of each microstate in MS. There is no significant difference between the two groups (high vs. low valence music stimuli). Each microstate has a similar probability of occurrence (max = 12.24, min = 7.76 for the high valence group). The probability of microstate #6 is relatively lower than others.

**Figure 5:**
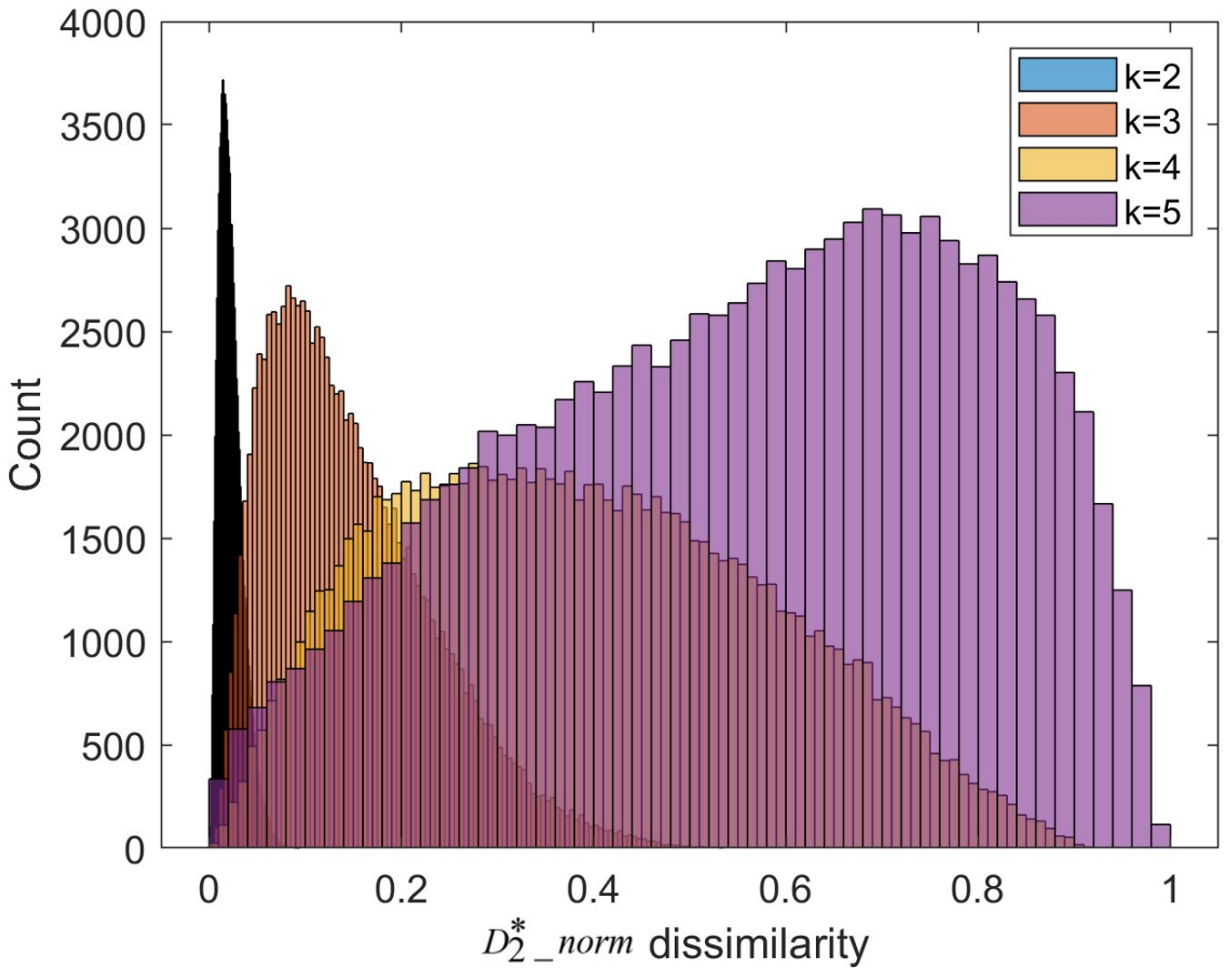
The $D_{2}^{*}\_norm$ dissimilarities distribution for different length of k-mer. It is worth noting that the color of histogram looks like black due to the over-concentrated distribution at *k* = 2.

We apply *k*-mer frequency analysis to analyze the MS. We set different lengths of *k*-mer (*k* = 2,3,4,5). For each k, we calculate the $D_{2}^{*}\_norm$ dissimilarity matrix between trials of the high valence group and trials of the low valence group. This matrix is symmetric. We plot the distribution of the upper triangular in Figure 5. When the length of *k*-mer is 2, the $10^{2}$ 2-mer can be considered as transitions between the single microstate. Its frequency is similar to conventional transition probabilities. As is shown in Figure 4, the dissimilarity of 2-mer between the high vs. low valence groups is low. Previous research suggests transition probabilities cannot model transition dynamics for longer time series of at least several minutes (von Wegner et al., 2017). When increasing the parameter k from 2 to 5, the larger dissimilarity between the two groups can be observed.

### Recognition results

**Figure 6:**
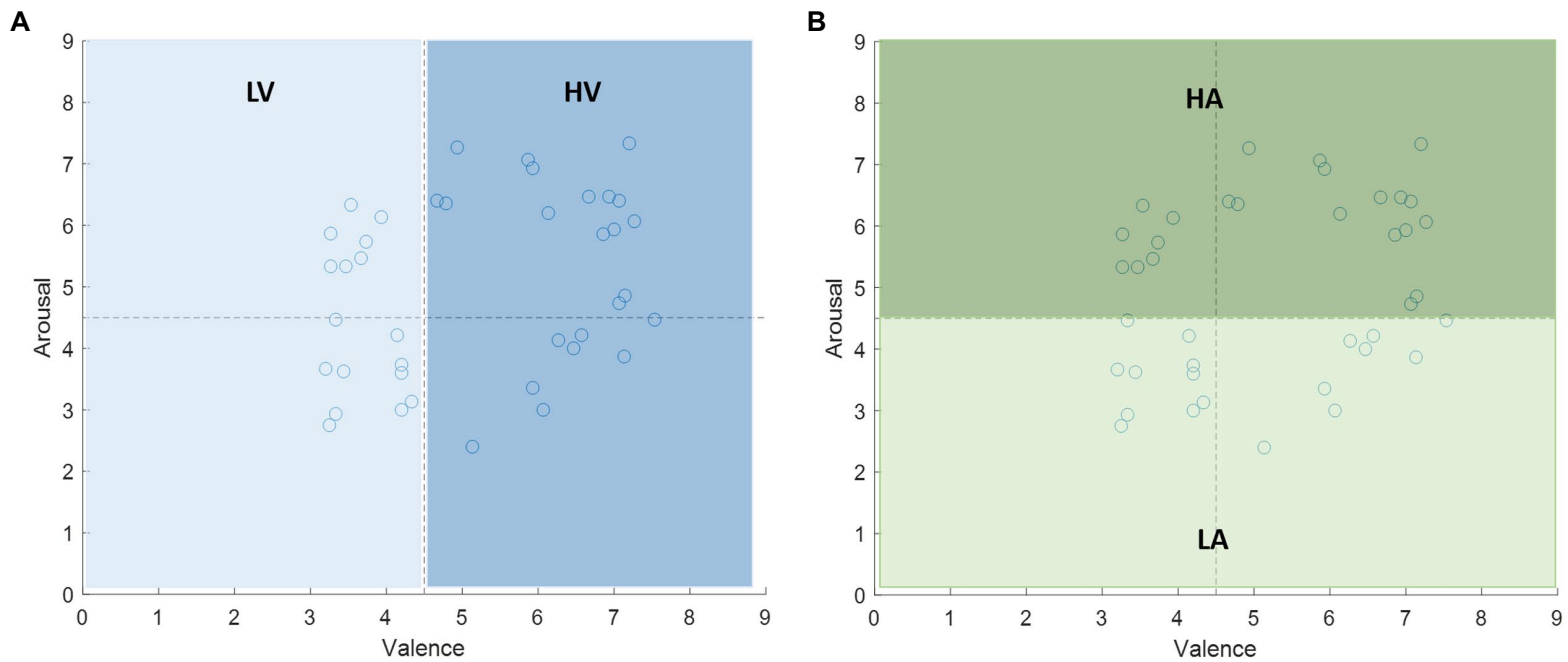
The labels of the stimuli on the valence-arousal plane. The median 4.5 is the threshold to divide the rating value into two categories: **(A)** low valence (LV) and high valence (HV) in valence dimension, **(B)** low arousal (LA) and high arousal (HA) in arousal dimension.

The performance of the proposed features for EEG-based emotion recognition is studied in this subsection. We evaluate the prediction accuracy on the DEAP dataset. We extract the 60s preprocessed EEG signals induced by a music video in each trial for each subject. The total number of EEG epochs from each subject was 40. Thus, the dimension of this dataset was 7680(time points)×32(channels)×40(epochs)×32(subjects) . For the emotional labels, each video has emotional rating values in the range of 1–9 in the arousal and valence domain. We set the 4.5 as the threshold to divide the rating value into two categories: more than 4.5 labeled with high arousal/valence, and less than or equal to 4.5 labeled with low arousal/valence. The labels are shown in Figure 6.

We use the 5-fold cross-validation method to increase the reliability of the classification results. The entire dataset was split into 5 folds. In each iteration, one fold (256 trials) is used to test the model and the rests (1,024 trials) serve as the training set. The process is repeated until each fold of the 5 folds has been used as the training set.

The criterion for evaluating the performance of two-class classification is accuracy:

(10) $$\mathrm{Accuracy}=\frac{TP+TN}{TP+TN+FN+FP}$$

where TP, TN, FP, and FN denote true positive, true negative, false positive, and false negative, respectively.

In this section, three experiments are conducted on the dataset. All experiments use SVM as a classifier. In the first experiment, we evaluate the performance of the fine-level features. The *k* of *k*-mer is set to 3,4,5. Here we use 10 microstates and we can construct a length of $10^{3},10^{4},10^{5}$ feature vector based on formula 9, respectively. The increase of k value will lead to exponential growth of the amount of computation. If four classical microstates are used, it is suggested to try a longer k-mer. PCA is used for dimension reduction. The number of components is selected based on the variance. The selected components need to explain a 85% percentage of the variance. In the second experiment, we evaluate the performance of the coarse-level features. Four parameters (duration, occurrence, time coverage, GEV) of each microstate class are extracted as features. In the third experiment, the fusion of fine and coarse features is used as the feature set. As 5-mer performs better in the first experiment, features of 5-mer are used as fine features.

Table 1 provides the accuracy of SVM classifiers for the valence and arousal dimensions using different feature sets. As can be seen, the classification performance of 5-mer is better than 3-mer and 4-mer. The fusion features at the fine and coarse levels give a better performance than those based on an individual level. The accuracy with the combination of 5-mer and four conventional temporal features is 64.8% for valence and 61.4% for arousal. We compare our proposed features to the original study of the DEAP dataset and other studies. The results are given in Table 1. The original study used spectral power and the spectral power asymmetry between 14 pairs of electrodes. Mert and Akan (2018) proposed MEMD-based features where MEMD is a time-frequency analysis method. Clerico et al. (2015) proposed an inter-hemispheric amplitude-modulated interaction feature set (IAMI) for emotion recognition. Compared to IAMI features, our features are more successful for both valence and arousal dimensions. Compared with spectral power-related features, the accuracy of our features is improved by 7% for the valence dimension, whereas it is reduced by 0.6% for the arousal dimension. Compare with MEMD-based features, the accuracy of our features is improved by 10% for the arousal dimension, whereas it is reduced by 2.2% for the valence dimension. By comparison, our proposed features are effective temporal features for EEG-based emotion recognition. In addition, it is suggested that feature extraction based on microstate sequences of different frequency bands should be considered in future work.

**Table 1:** The classification accuracies of different feature sets on DEAP dataset.

Study	Features	No. features	Valence	Arousal
Koelstra et al. (2011)	Spectral power, the spectral power asymmetry between 14 pairs of electrodes	216	57.6%	62.0%
Clerico et al. (2015)	IAMI^a^ feature sets	115(arousal) 53 (valence)	61%	61%
Mert and Akan (2018)	MEMD-based features	250(after ICA)	67.00%	51.01%
Ours-fine level	F𝑤 of 3-mer	15	57.3%	56.3%
	F𝑤 of 4-mer	23	59.1%	57.8%
	F𝑤 of 5-mer	39	60.9%	60.1%
Ours-coarse level	TF^b^	40	62.7%	59.9%
Ours-fine and coarse level	F𝑤 of 5-mer, TF^b^	79	64.8%	61.4%

^a^IAMI, inter-hemispheric amplitude modulated interaction feature; ^b^TF (temporal features), duration, occurrence, time coverage, GEV.

## Conclusion

Emotion recognition based on EEG has received increased attention in the field of affective computing. In this study, we propose a novel feature extraction method for emotion recognition. First, a microstate sequence is obtained using microstate analysis. Then, we extract features of the microstate sequence at fine and coarse levels. At the fine level, we propose a feature set derived from the statistics of k-mer. At the coarse level, we extract conventional four temporal parameters. Finally, SVM is used as a classifier for emotion recognition. These features are evaluated on the DEAP dataset. The classification results demonstrate that the fusion of fine and coarse features gives a better performance than those based on an individual level. Compared with other features, our proposed features are effective temporal features for EEG-based emotion recognition. In addition, it is suggested that feature extraction based on microstate sequences should consider different frequency bands in future work.

## Data availability statement

The original contributions presented in the study are included in the article/supplementary material, further inquiries can be directed to the corresponding authors.

## Author contributions

JC and ZZ were involved in the conduct of the experiment, data analysis and writing of the manuscript. QS was involved in the conception, supervision. GC was involved in the conception, review and revision. All authors contributed to the article and approved the submitted version.

## Funding

Key projects of Zhejiang Provincial Department of Culture and Tourism jointly built by provinces and ministries (2022518921).

## Conflict of interest

The authors declare that the research was conducted in the absence of any commercial or financial relationships that could be construed as a potential conflict of interest.

## Publisher’s note

All claims expressed in this article are solely those of the authors and do not necessarily represent those of their affiliated organizations, or those of the publisher, the editors and the reviewers. Any product that may be evaluated in this article, or claim that may be made by its manufacturer, is not guaranteed or endorsed by the publisher.

## References

[ref1] Al Zoubi O. Mayeli A. Tsuchiyagaito A. Misaki M. Zotev V. Refai H.. (2019). EEG microstates temporal dynamics differentiate individuals with mood and anxiety disorders from healthy subjects. Front. Hum. Neurosci. 13:56. doi: 10.3389/fnhum.2019.00056, PMID: 30863294 PMC6399140

[ref2] Balasubramanian G. Kanagasabai A. Mohan J. Seshadri N. G. (2018). Music induced emotion using wavelet packet decomposition—an EEG study. Biomed. Signal Process. Control 42, 115–128. doi: 10.1016/j.bspc.2018.01.015

[ref3] Chen J. Li H. Ma L. Bo H. Soong F. Shi Y. (2021). Dual-threshold-based microstate analysis on characterizing temporal dynamics of affective process and emotion recognition from EEG signals. Front. Neurosci. 15, 689791. doi: 10.3389/fnins.2021.689791 34335165 PMC8318040

[ref4] Chen J. Li H. Ma L. Soong F. (2022). DEEMD-SPP: a novel framework for emotion recognition based on EEG signals. Front. Psychiatry 13, 885120. doi: 10.3389/fpsyt.2022.885120 35573327 PMC9091650

[ref5] Clerico A. Gupta R. Falk T. H. (2015). *Mutual Information between Inter-hemispheric EEG Spectro-temporal Patterns: A New Feature for Automated Affect Recognition*. Paper presented at the 2015 7th international IEEE/EMBS conference on neural engineering (NER).

[ref6] D’Croz-Baron D. F. Baker M. Michel C. M. Karp T. (2019). EEG microstates analysis in young adults with autism spectrum disorder during resting-state. Front. Hum. Neurosci. 13:173. doi: 10.3389/fnhum.2019.00173, PMID: 31244624 PMC6581708

[ref7] Daly I. Malik A. Hwang F. Roesch E. Weaver J. Kirke A.. (2014). Neural correlates of emotional responses to music: an EEG study. Neurosci. Lett. 573, 52–57. doi: 10.1016/j.neulet.2014.05.003, PMID: 24820541

[ref8] Deorowicz S. (2020). FQSqueezer: k-mer-based compression of sequencing data. Sci. Rep. 10, 1–9. doi: 10.1038/s41598-020-57452-6 31953467 PMC6969201

[ref9] Gianotti L. R. Faber P. L. Schuler M. Pascual-Marqui R. D. Kochi K. Lehmann D. (2008). First valence, then arousal: the temporal dynamics of brain electric activity evoked by emotional stimuli. Brain Topogr. 20, 143–156. doi: 10.1007/s10548-007-0041-2, PMID: 18175212

[ref10] Hsu J.-L. Zhen Y.-L. Lin T.-C. Chiu Y.-S. (2018). Affective content analysis of music emotion through EEG. Multimedia Systems 24, 195–210. doi: 10.1007/s00530-017-0542-0

[ref11] Hu W. Zhang Z. Zhang L. Huang G. Li L. Liang Z. (2022). Microstate detection in naturalistic electroencephalography data: a systematic comparison of topographical clustering strategies on an emotional database. Front. Neurosci. 16, 812624. doi: 10.3389/fnins.2022.812624, PMID: 35237121 PMC8882921

[ref12] Jenke R. Peer A. Buss M. (2014). Feature extraction and selection for emotion recognition from EEG. IEEE Trans. Affect. Comput. 5, 327–339. doi: 10.1109/TAFFC.2014.2339834

[ref13] Kim K. Duc N. T. Choi M. Lee B. (2021). EEG microstate features for schizophrenia classification. PLoS One 16:e0251842. doi: 10.1371/journal.pone.0251842, PMID: 33989352 PMC8121321

[ref14] Kirk J. M. Kim S. O. Inoue K. Smola M. J. Lee D. M. Schertzer M. D.. (2018). Functional classification of long non-coding RNAs by k-mer content. Nat. Genet. 50, 1474–1482. doi: 10.1038/s41588-018-0207-8, PMID: 30224646 PMC6262761

[ref15] Koelstra S. Muhl C. Soleymani M. Lee J.-S. Yazdani A. Ebrahimi T.. (2011). Deap: a database for emotion analysis; using physiological signals. IEEE Trans. Affect. Comput. 3, 18–31. doi: 10.1109/T-AFFC.2011.15

[ref16] Lehmann D. Ozaki H. Pál I. (1987). EEG alpha map series: brain micro-states by space-oriented adaptive segmentation. Electroencephalogr. Clin. Neurophysiol. 67, 271–288. doi: 10.1016/0013-4694(87)90025-3, PMID: 2441961

[ref17] Lin Y.-P. Wang C.-H. Jung T.-P. Wu T.-L. Jeng S.-K. Duann J.-R.. (2010). EEG-based emotion recognition in music listening. IEEE Trans. Biomed. Eng. 57, 1798–1806. doi: 10.1109/TBME.2010.2048568, PMID: 20442037

[ref18] Marçais G. Kingsford C. (2011). A fast, lock-free approach for efficient parallel counting of occurrences of k-mers. Bioinformatics 27, 764–770. doi: 10.1093/bioinformatics/btr011, PMID: 21217122 PMC3051319

[ref19] Mert A. Akan A. (2018). Emotion recognition from EEG signals by using multivariate empirical mode decomposition. Pattern. Anal. Applic. 21, 81–89. doi: 10.1007/s10044-016-0567-6

[ref20] Michel C. M. Koenig T. (2018). EEG microstates as a tool for studying the temporal dynamics of whole-brain neuronal networks: a review. NeuroImage 180, 577–593. doi: 10.1016/j.neuroimage.2017.11.062, PMID: 29196270

[ref21] Murray K. D. Webers C. Ong C. S. Borevitz J. Warthmann N. (2017). kWIP: the k-mer weighted inner product, a de novo estimator of genetic similarity. PLoS Comput. Biol. 13:e1005727. doi: 10.1371/journal.pcbi.1005727, PMID: 28873405 PMC5600398

[ref22] Muthukrishnan S.-P. Ahuja N. Mehta N. Sharma R. (2016). Functional brain microstate predicts the outcome in a visuospatial working memory task. Behav. Brain Res. 314, 134–142. doi: 10.1016/j.bbr.2016.08.020, PMID: 27515287

[ref23] Pascual-Marqui R. D. Michel C. M. Lehmann D. (1995). Segmentation of brain electrical activity into microstates: model estimation and validation. IEEE Trans. Biomed. Eng. 42, 658–665. doi: 10.1109/10.391164, PMID: 7622149

[ref24] Reinert G. Chew D. Sun F. Waterman M. S. (2009). Alignment-free sequence comparison (I): statistics and power. J. Comput. Biol. 16, 1615–1634. doi: 10.1089/cmb.2009.0198, PMID: 20001252 PMC2818754

[ref25] Salas C. E. Radovic D. Turnbull O. H. (2012). Inside-out: comparing internally generated and externally generated basic emotions. Emotion 12, 568–578. doi: 10.1037/a0025811, PMID: 22023364

[ref26] Shen X. Hu X. Liu S. Song S. Zhang D. (2020). *Exploring EEG Microstates for Affective Computing: Decoding Valence and Arousal Experiences during Video Watching*. Paper presented at the 2020 42nd Annual International Conference of the IEEE Engineering in Medicine & Biology Society (EMBC). 10.1109/EMBC44109.2020.9175482 33018116

[ref27] Song T. Zheng W. Song P. Cui Z. (2018). EEG emotion recognition using dynamical graph convolutional neural networks. IEEE Trans. Affect. Comput., 11, 532–541.

[ref28] Suhaimi N. S. Mountstephens J. Teo J. (2020). EEG-based emotion recognition: a state-of-the-art review of current trends and opportunities. Comput. Intell. Neurosci. 2020, 1–19. doi: 10.1155/2020/8875426 PMC7516734 33014031

[ref29] Tao W. Li C. Song R. Cheng J. Liu Y. Wan F.. (2020). EEG-based emotion recognition via channel-wise attention and self attention. IEEE Trans. Affect. Comput. doi: 10.1109/TAFFC.2020.3025777

[ref30] Torres E. P. Torres E. A. Hernández-Álvarez M. Yoo S. G. (2020). EEG-based BCI emotion recognition: a survey. Sensors 20:5083. doi: 10.3390/s20185083, PMID: 32906731 PMC7570756

[ref31] von Wegner F. Tagliazucchi E. Laufs H. (2017). Information-theoretical analysis of resting state EEG microstate sequences-non-Markovianity, non-stationarity and periodicities. NeuroImage 158, 99–111. doi: 10.1016/j.neuroimage.2017.06.062, PMID: 28673879

[ref32] Wan L. Reinert G. Sun F. Waterman M. S. (2010). Alignment-free sequence comparison (II): theoretical power of comparison statistics. J. Comput. Biol. 17, 1467–1490. doi: 10.1089/cmb.2010.0056, PMID: 20973742 PMC3123933

[ref33] Wen J. Chan R. H. Yau S.-C. He R. L. Yau S. S. (2014). K-mer natural vector and its application to the phylogenetic analysis of genetic sequences. Gene 546, 25–34. doi: 10.1016/j.gene.2014.05.043, PMID: 24858075 PMC4096558

